# Making grandchildren. Is there an interest in becoming a grandparent?

**DOI:** 10.1007/s11019-025-10311-5

**Published:** 2025-11-25

**Authors:** Daniela Cutas

**Affiliations:** Lund University, Lund, Sweden

**Keywords:** Interest in becoming a parent, Interest in becoming a grandparent, Grandparenthood, Grandparents, Grandchildren, Intrafamilial reproduction

## Abstract

In recent decades, with the advancement of technologies facilitating reproduction, parents have been able to make decisions regarding their childrens’ reproductive potential: to preserve their fertility when threatened by genetic conditions or medical treatment; to use their gametes or embryos to make grandchildren; or even to collect their gametes posthumously and then use them to make grandchildren. While these interventions tend to be framed in terms of the interests of the children themselves, parents are not indifferent as to whether they become grandparents. At the same time, while the interest in becoming a parent and parents’ interests have been discussed extensively in reproductive and family ethics, grandparents and grandparenthood have been at best marginal in this growing literature. Against this background, I ask the question whether parents have an interest in becoming grandparents – and if so, which claims this interest generates against other parties (such as children themselves, healthcare professionals, or the state), if any. I explore possible paths towards conceptualising such an interest and the degree to which it could ground a claim to facilitate its fulfilment.

## Introduction

I start my analysis by offering a number of examples of cases in which parents have been involved in their children’s reproduction: these range from interventions meant to safeguard children’s reproductive potential, to parents making grandchildren themselves. I subsequently explore how an interest in becoming a grandparent could be substantiated, and what it may require. Finally, I outline some challenges for such an interest. I aim to show that there is a need for a more explicit analysis of intrafamilial interests that takes into account *all* the forces that may do substantial normative work in this area, including the, often implicit, wishes and interests of prospective grandparents.

People who wish to become parents can take measures to do so. They can seek a like-minded partner to become a parent with. They can aim to become single parents and avail themselves of gamete donation or assisted reproduction in order to achieve this. Either way, unilaterally deciding to become a parent and fulfilling one’s wish are never completely solo endeavours. Some kind of cooperation is required from at least one other person (or a group of participants in the case of assisted reproduction).

A wish to become a grandparent is however typically doubly dependent on others’ wishes: the prospective grandparent’s existing children, and someone else whose reproductive material and/or reproductive potential is also necessary – either as a gamete donor, surrogate mother, or simply the grandchild’s other parent. In contrast to the wish to become a parent, in the Western world, it tends to not be regarded as acceptable for people to openly pursue their grandchild wish. It is only up to the prospective parents themselves if they wish to become parents, and there is nothing that those wishing to become grandparents can do to make it happen (other than mention it many times to their children!). With medical technology, however, parents *can* and sometimes *do* take steps that make grandparenthood possible for them, or even set events into motion to make their own grandchildren.

Obviously, the first step to making grandparenthood possible is to become a parent. But sometimes one’s child may be at risk of losing their fertility while young – or lose their life before they become parents. Family members of children or adults may in some legislatures authorise or require the collection, storage, transfer, or use of their children’s reproductive material.

For example, parents can arrange the collection and storage of reproductive material from their children who are at risk of losing their fertility because of certain genetic conditions or medical treatments (which may compromise the patient’s fertility). This is done ostensibly in the (future) interest of the child, so that they can reproduce biologically when they are adults (Hens and Cutas 2015). However, when they are interviewed, the parents sometimes also give hints of their own hopes about their children’s reproduction and parenthood. In one study, parents of children at risk to become infertile as adults referred to the prospect of their children’s infertility as a “key concern” (Johnson et al. 2017). Researchers having interviewed such parents remarked that the interviewees seemed to be “vicariously and in an anticipatory way going through a ‘crisis of infertility’ involving themselves and their child” (p. 218). These parents experienced distress because of this to such an extent that researchers worried about the impact of this on the child’s self-image in the future (Borelli et al. 1984). Some researchers expressed concern that parents’ attitudes towards the prospect of their children’s infertility risk reinforcing norms and expectations about women and childbearing or men, masculinity and fertility (Nisker et al. 2006). Another research team commented on their interviewees’ feelings and attitudes as congruent with an experience of “grief related to the loss of potential grandchildren” (Bourke et al. 2014). Once fertility preservation measures have been taken, there is also a risk of “steering children’s future towards child bearing” and pressure to use the material that the parents have preserved (Dolin et al. 2010). Therefore, while fertility preservation for children is in principle undertaken for the children’s sake, empirical studies suggest that the parents’ own projections and suffering can also be significant in their decision-making.

This suffering seems relevant in other contexts as well. Armed conflict takes many soldiers’ lives, and the families of some of those soldiers express interest in collecting reproductive material from them and using it to make children. In Israel, sperm can be collected postmortem from deceased soldiers at their parents’ request and used in reproduction. Most posthumous sperm retrieval interventions since the start of the war have been initiated by the parents of the deceased (Savitsky 2025). One media account of this practice referred to it as parents “fighting for the right to have grandchildren” (Callanan 2024). A parent is quoted as stating that “when his son was killed, he [the son] was ‘stopped from being a parent’ but he [the parent] does not want to be ‘stopped from being a grandparent’” (Dawson 2022). In examples such as these, the wishes of the prospective grandparents are explicit as they pursue what has been called “posthumous grandparenthood” (Hashiloni-Dolev 2018).

These are also other ways in which prospective grandparents have contributed towards making grandparenthood possible. Many cases of uterus donation have involved the transfer of a uterus from a mother to her daughter. In Sweden, most of the first women who underwent uterus transplants saw their mothers as the most obvious donors (Guntram 2021). Mothers have ‘lent’ their uterus to their children long before uterus transplants became a thing: for example, in 2015, a man became the father of a baby whose birth mother was his own mother (EWFC 2015), in a case of familial surrogacy. These practices create new connections in which the parents, and especially the mothers, are the means by which their children reproduce. As noted, ostensibly this is done in support of the prospective parents but it also happens to facilitate one’s own grandparenthood aspirations. Is this consideration something that could have played a role, at least in some of these cases?

## What is it to have an interest?

In this paper, I refer to ‘interests’, ‘rights’, and ‘wishes’. These concepts have been used, sometimes interchangeably, in the reproductive and family ethics literature. However one defines them, it seems salient that rights and interests must be more significant than ‘mere’ wishes. Whether people have a claim on others to support them in their reproductive or parenting endeavours must hinge on more than their being interested in it – or having a so-called ‘child wish’. Interests tend to be described as more substantial than wishes: an interest may generate a claim on another party, whereas a wish may not. Moreover, one may have an interest even if one is not interested in the object of that interest – or may not ‘wish’ it (Brighouse and Swift 2014; p. 52). I may have an interest in something in the sense that it would be good for me, whether or not I am interested in it. In some interpretations (notably interest theories of moral rights, very influential in family ethics), an interest can ground a right. A wish will have far less normative bite – though it can still be action guiding insofar as being free to pursue one’s own goals is an important tenet of liberalism.

While there may be good reasons to avoid voicing an interest in becoming a grandparent when making decisions regarding the reproductive potential of one’s child, it seems that there are also reasons to suspect that such an interest may be at play in some of these cases. Both meanings of ‘interest’ can be at play here: prospective grandparents may be interested in their grandparenting future, *and* they may have an interest in becoming grandparents which grounds a claim on others. An interest in the former sense (of being interested) may not by itself suffice to ground any substantial normative claim. In the following, I aim to discuss what an interest in the latter sense could be and which kinds of claims it could ground – if any.

## Could there be an interest in becoming a grandparent?

Intuitively, it seems straightforward that there can be a wish to become a grandparent and have a grandparent-grandchild relationship. Many parents look forward to having grandchildren, and when they do become grandparents, they deeply appreciate the relationships that they build with their grandchildren. Some may be more involved as grandparents than they were as parents. They may be less stressed than they were when they were younger, having already built their career. They may have more financial stability and emotional maturity than they did when they were raising their own children. Grandparenthood may bring about many of the relationship goods that people can derive from parenthood, but with fewer of the burdens. (That grandparenthood presupposes fewer burdens is, of course, not always the case. There are many instances in which grandparents take on most or all responsibilities for their grandchildren, temporarily or permanently^1^, and this is more common in some cultures than in others.)

Reporting the results of a longitudinal study undertaken in the 1990s of 300 grandparents and grandchildren, Kornhaber referred to “an organically based grandparent drive”, which is “similar to the drive to have a child – in other words, it springs from the impulse to reproduce, establish a generational continuum, protect and nurture the species, and love and care for a child born from the self” (Kornhaber 1996).

Interestingly, all the parts of this account of the ‘grandparent drive’ hinge on the biological connection between (grand)parent and (grand)child: the grandparent drive is about reproduction, establishing a continuum, the species, and the self (from which the grandchild originates via the parent). Much has happened since 1996, and the prominence of the biological or more specifically genetic continuation aspect of (grand)parent-(grand)child relationships has lost much of its centrality. Instead, the focus has been shifted to the quality of family relationships (Golombok 2020), and the relationship goods that these can facilitate for family members (Gheaus et al. 2018). While it is plausible that, for many, genetic ties are still at the core of their conception of family and family relationships, it has become less obvious that it must be so. Moreover, in many of the cases in which parents take measures that make grandparenthood possible for them, directly (by creating grandchildren) or indirectly (by taking fertility preservation measures on behalf of their children), we do not know whether the parents and their children are genetically related. They may not be, and the parents may still value genetic relatedness between their children and grandchildren.

Kornhaber himself allows for a conception of grandparenthood that is not necessarily dependent on biology: grandparenting “is a *social function* of elders linked to life meaning and satisfaction and to *social usefulness*” (Kornhaber 1996) (my emphasis). This perspective can help make sense of some of the ways in which grandparenthood is a good for the prospective grandparents, but also for society as a whole.

Prospective grandparents may be at greater risk of social deprivation than prospective parents. They may be empty nesters and no longer employed, may have more restricted social circles, and may have a harder time finding a new partner or forging new close personal relationships. They may have fewer opportunities to engage with others of different ages. Philosophers have argued that the costs of social deprivation ground a duty to create opportunities for people to have their social needs met (Brownlee 2020). People’s adult children are often likely to be better placed than others to respond to their own parents’ social needs: and the company of grandchildren may be an excellent way to meet this need *and* provide cross-generational meaningful interaction to all parties involved.

Grandchildren can contribute to their grandparents’ identity and sense of self not only by being children and thus acquainting their grandparents with the world from a child’s perspective in the present, but also by doing so in the context of being raised by their parents (Overall 2022). When this happens within a well-functioning family, it can offer the grandparents opportunities for personal growth that are unavailable by any other means.

It should also be noted here that prospective grandparents are not the same social group as potential parents. They (presumably) already chose to become parents: not all adults do. They may therefore be more likely than the general population to care about playing a nurturing role for children. They care about parenting, and they care about their own children: their grandchildren’s parents. Insofar as they care about ‘generational continuums’ or nurturing the species or creating children from the self, they may care about their own children not breaking this chain.

But how could we conceptualise an interest in becoming a grandparent?

One way of doing this is to look at how the interest in becoming a parent has been conceptualised. One particularly influential account starts with the importance of intimate relationships in one’s life (Brighouse and Swift 2014). Meaningful intimate relationships can be of several kinds, and each of these kinds can be enriching in their own way. The parent-child relationship

“involves the adult in a unique combination of joys and challenges; experiencing and meeting these makes a distinctive set of demands on him, and produces a distinctive contribution to his well-being. Other intimate relationships have their own value, but they are not substitutes for a parenting relationship with a child” (p. 88).

Parents’ rights are conditional on the interests of their children, and children have an interest in having parents who will take care of them and promote their interests. Brighouse and Swift describe this relationship, and the interplay between parents’ and children’s interests, as ‘unique’. Grandparents or parents’ friends may also be close to children, but they ultimately are not those who have primary responsibility for them nor authority for deciding how this responsibility will be exercised. This is what makes the parent-child relationship unique (p. 93).

Brighouse and Swift clump grandparents together with friends, after acknowledging that different kinds of intimacy contribute to children (and others’) interests in different ways. But the grandparent-grandchild relationship has some parent-child relationship built in. After all, a grandparent, in the common sense of the word, is the parent’s parent. Furthermore, that it is the parent and not the grandparent who has primary responsibility is a social and legal convention, and is not universal either in time or space. In some cultures, responsibility for children is shared within the community (Fayemi 2017). In others, grandparents are the heads of the household in which the grandchildren are raised (Kligman 1988) or may be the primary caregivers for grandchildren’s first and most formative years, and sometimes until adulthood (Pantea 2011). Therefore, although the parent-child relationship is very important, all of the roles that we tend to associate with parenthood do not necessarily coincide in the same one or two individuals by default – especially not in a way that separates all parents from all other caregivers.

One could note here that if a grandparent performs the parenting role, then they are effectively a parent, and so in such cases theirs is a parent-child rather than grandparent-grandchild relationship. This may ultimately hinge on how we define our terms. If indeed a grandparent is a parent’s parent, then the grandchild-raising grandparent is both a parent and a grandparent. Likewise, the grandparents involved in some of the examples mentioned above effectively become the parents of their grandchildren. In the end these distinctions may simply be superfluous, and we may need to acknowledge that there is fluidity between some of the intimate roles that people play in children’s lives. Before the end of the paper, I will revisit this relation between parenthood and grandparenthood.

All of the above suggests that there may well be an interest in becoming a grandparent: like the parent-child relationship, the grandparent-grandchild relationship can be deeply meaningful for both the grandparent and the grandchild. However, if we are to borrow and adjust Brighouse and Swift’s framework, any ‘right’ that a parent may have to grandparent also needs to be conditional on the interests of children. Can we make that argument?

Like adults, and maybe more than adults, children also have an interest in relating to people other than, and in addition to their parents. Gheaus, for example, has argued not only that individuals who are not the parents should be involved in providing care for children, but that care for all children should be shared between parents and others (Gheaus 2011). However good a child’s parents may be at parenting her, nonparental care is important even in those cases in order to avoid monopolies of care and mitigate children’s vulnerability to their primary carers (p. 487, see also Shields forthcoming).

If we accept that children have an interest in having carers who are not their parents, there are many reasons why grandparents are good candidates for this role. They (presumably) know and love the children’s parents. They may have more free time and more patience than the parents to relate to the child and to engage in children’s favourite activities and to ‘spoil them’ while the parents might feel more constrained by their responsibilities to be cautious or stern. Grandparents can teach children about other life stages and share life experiences that the parents may not have yet experienced or may not have the time or the inclination to ponder due to being in the middle of both childrearing and career building. The wider age gap in itself may contribute something that the parents or others closer in age to the child cannot. Grandparents can be a link to other family members beyond the children’s nuclear family. And so on.

Writing about the age gap specifically, Overall builds on empirical data to show that grandparenthood can be beneficial to a child’s sense of identity, understanding of recent history and broader family ties including affective ties to earlier generations. Children can also gain a new perspective on their own parents as children, though the grandparents’ eyes. Grandparents’ experience of parenting the children’s parents can also make them well placed to mediate between the children and the parents – and Overall cites empirical data in the US that suggests that almost half of grandparents contribute this kind of mediation (p. 149).

In sum, there are reasons to acknowledge both that there may be an interest in being a grandparent, and an interest in being grandparented. A good case can be made, based on considerations such as those listed above, in favour of both these interests. But if we accept that there are such interests, what follows from this?

## If there is an interest in becoming a grandparent, which claims does it ground?

It seems obvious that where a grandparent-grandchild relationship has already been established, it can be deeply harmful to both parties that it be broken. This is increasingly acknowledged as courts recognise grandparent-grandchild bonds and prevent parents from severing them (Henderson 2005).

It has however been argued that grandparents qua grandparents are not entitled either to form or to continue relationships with their grandchildren (Draper 2013). While it may be in the interest of a child to continue a relationship with their grandparent, this is not *because* the adult is their grandparent, but because the relationship is close. And, Draper argues, if a grandparent effectively becomes a child’s parent, then they may acquire rights and duties in relation to the child because of their parental role.

However, as Brighouse and Swift note, close personal relationships (or meaningful intimate relationships, in the authors’ words) are not substitutable with each other. While of course there will be overlaps, the relationship paths between a child and a family friend, a neighbour, or a grandparent are very different. There is a presumption of a continuity of care between parents and their children and their children’s children that may differ from the presumptions of other relationships. Parents may have responsibilities for their adult children that differ from those of other intimates. Children are a part of a family story in ways that are different from, for example, a friendship. As Overall notes, if a child who is your friend loses interest in the friendship, then they may cease to be your friend: but your grandchild is still your grandchild even if they lose interest in you (p. 155). In short, close personal relationships may not be substitutable with each other, the grandparent-grandchild relationship may have properties that other relationships do not have, and it may be very important for both grandparent and grandchild. If we grant all this, what follows?

In her paper, Draper looks specifically at grandparental *entitlements* and *obligations*. The matter of whether there is an interest in *becoming* a grandparent is separate from these questions. I may have an interest and at the same time there may be reasons why this interest cannot generate duties for others. Not all interests are equal, and some interests that we have may conflict with weightier interests. Unlike the interest in becoming a parent, which is recognised in human rights instruments, or even the interests in maintaining an already formed grandparent-grandchild relationship, it seems less obvious that there is anything that prospective grandparents can do to become grandparents, or that they can expect others to make them grandparents. In practice, as we have seen, developments in reproductive technology do sometimes offer routes towards grandparenthood and there are cases where people do choose them. However, these choices can be rife with potential clashes. When a parent takes steps to preserve their children’s fertility and thus keep their own grandparenting future open, there may be a conflict between their interest in furthering their own grandparenthood aspirations, and the interests of their children. There may also be a conflict between the children’s present interests (in survival, bodily integrity, privacy, etc). and their future, presumed interest in becoming biological parents (the latter of which the parents are trying to safeguard in taking fertility preservation measures, possibly at the expense of the former). In the case of dying or deceased adults, there may likewise be conflicts between the prospective grandparents’ interest in grandparenthood and dead or dying people’s interests in bodily integrity or privacy – as well as broader societal tabus around harvesting of the bodies of the deceased for personal and unauthorised reasons. Granted, postmortem organ donation is practiced with presumed consent in some legislatures, but in those cases the donation tends to be impersonal and not to the direct benefit of those who mandate it and control its use (Smajdor 2015).

## What is a grandparent?

It may seem awfully late, in the middle of the paper, to ask this question. But whether or not there can be an interest in becoming a grandparent, not everyone who seeks to or succeeds in becoming one are seeking, or getting, the same thing. In day-to-day life, in legal contexts, and in philosophical literature, people may mean different things when they say that someone is someone else’s parent. They may mean genetic parent: this is the person whose gametes have contributed to reproduction. They may mean biological parent: this is the birth mother. They may mean legal parent: these are the people who are legally recognised as a specific child’s parents. Legal parents may but need not be children’s biological or genetic parents. One may, in a court of law, be nominated a legal parent on the basis of the relationship that one already has with a child: the adult is acting as the child’s parent, and the child is attached to them. The question “who is Johnny’s parent” may therefore have different answers, depending on what we want to know more specifically.

Are there also several perspectives on what a grandparent is?

In a popular Swedish film, *Kan du vissla Johanna*^2^, Berra, a boy who has no grandparents, decides to find himself one, because he wants to experience a grandchild-grandparent relationship. He goes to a home for senior people and finds a nice man there, Nils, who is happy to pretend to be his grandfather. The boy and the man then develop a close relationship in which they effectively act as grandson and grandfather. Could we say that they have thus *become* grandson and grandfather?

Probably not. Although their close relationship may be very valuable to both of them, the man cannot be the boy’s grandfather because he is not the father of either of the boy’s parents. Their relationship, although it looks a lot like a grandparent-grandchild relationship, is not a grandparent-grandchild relationship, because of this. But let’s say that instead of being found by Berra, Nils is contacted by another person, who discovered that Nils is his gamete donor’s biological parent. Let’s further assume that Berra was not even aware that he had a biological child. Is Nils the grandparent of *this person*, in any sense of the word? It seems that he is. If both take a direct-to-consumer DNA test, the result will suggest that their genetic relationship is most likely that of grandparent/grandchild.

Empirical researchers are used to mismatches between whether people see themselves, or are seen by others, as grandparents, depending on whether they are genetically grandparents. The parents of gamete donors may express interest in their biological grandchildren, and conversely, grandparents in families conceived with donor gametes may not see the children as fully their grandchildren (Nordqvist and Smart 2014). One man having met children conceived with gametes donated by his son is quoted in a study as saying that he “went from zero to grandfather faster than anyone ever” (Hertz and Nelson 2019). So there is a sense in which one can become a grandparent in absence of a grandparent-grandchild close relationship.

Conversely, one can of course also become a grandparent in absence of close genetic ties between grandparent and grandchild *and* parent and child. Say, John adopts Sam who later adopts Mary. John is Mary’s grandfather, even if not in a straightforward biological sense.

How is all this relevant?

There are paths towards social grandparenthood that are not available in absence of a biological *or* close personal relationship between all three generations. But then it seems that genetic ties alone suffice for the recognition of a genetic grandparent-grandchild relationship in *one* sense, with no need for the succession of a close personal relationship between child, parent and grandparent. The goods most specific to grandparenthood, in Overall’s account, are not applicable in such a case: the child is not being raised by their parent, who was raised by the grandparent. This also means that while Berra and Nils are contributing to each other’s wellbeing in substantial ways that make their relationship very valuable to both, these are not the ways that are specific to the grandparent-grandchild relationship. This would also be the case should it turn out that, unbeknownst to them both, Berra is in fact Nils’ biological grandchild.

But this also can provide an answer to the question whether an interest in grandparenthood can be served simply by facilitating grandparent-grandchild-like relationships between children who do not have grandparents and adults who do not have grandchildren. While these relationships can be very valuable, they do not address all of the interest. Therefore, the interest cannot be said to be fulfilled unless the grandparent is the rearing parent of the rearing parent, at least in line with Overall’s account. This being the case, there may still be good reasons to encourage and support these close relationships, because of all the goods that *can* be developed in them, some of which coincide with the goods in other close relationships, including the grandparent-grandchild relationship. This is analogous to the way in which spending a lot of time with children may be beneficial to a person who longs to become a parent, but it cannot replace a parent-child relationship.

At the same time, ‘grandparenthood’ is sometimes a misnomer. Parents who decide to make children using their children’s reproductive material also do not experience the typical grandparent/grandchild relationship that Overall describes. In some cases, they effectively become the children’s parents, in at least some common understandings of parenthood. A woman who gives birth to her daughter’s child, as in familial surrogacy, is the child’s birth mother. The parents of a deceased soldier whose gametes they used to create a child may raise that child themselves as the legal parents. Or they may have the child raised by someone else and relate to her as her grandparents. These are not the specific kind of intergenerational relationships that Overall had in mind. Therefore, an attempt to ground an interest in becoming a grandparent in these cases using Overall’s analysis is at least insufficient.

Overall’s case for the goods of grandparenthood, while unspecific regarding genetic ties, is quite specific regarding social relationships. The interest in grandparenthood, in line with this account, is based on social needs and interpersonal relationships in a certain succession. Therefore, it may work more straightforwardly to support a claim to being allowed to play the social role of a grandparent to one’s already existing grandchildren than it does in making grandchildren. Analogously, while there may be an interest in becoming a parent, this interest does not by itself ground a claim to be a parent. Instead, as noted above, the interest in parenting a certain child must be correlated with the interest of the child in having a parent (Brighouse and Swift 2014). But maybe the interest in grandparenthood does ground a claim to be allowed to form and maintain a grandparent-grandchild relationship?

## Becoming and being a grandparent

While they both write about grandparenthood, Overall and Draper write about different aspects of it and also take different perspectives. Draper writes from the perspective of children’s interests. Overall writes from the perspective of the interests that people might have in experiencing a grandparent-grandchild relationship, both as children and as (prospective) grandparents.

When a close relationship has already been established, a claim to its continuation can be argued for from the perspective of the legitimate interests of both parties. A legal guardian’s unilateral decision to sever a bond already formed can predictably hurt the child. Depriving a child of the opportunity to develop a relationship that includes goods such as those described by Overall could also be detrimental to the child, though in a more subtle way: they have not yet developed an attachment to that specific person.

For both Draper and Overall, the relevant sense of grandparenthood is located in the relationship goods that grandparenthood can bring about (whether because it is grandparenthood, for Overall, or because it is a close relationship, for Draper). However, acting to preserve the possibility of becoming a genetic grandparent may be neither. Ensuring that one’s child can still become a genetic parent is one step towards becoming a grandparent. Should parents not take steps to preserve their children’s fertility, they can still become grandparents. Some parents may specifically value genetic grandparenthood. But the arguments in favour of an interest in grandparenthood discussed thus far do not hinge on the genetic relationship between any of the parties nor do they support the creation of genetic relationships specifically.

If there is an interest in having a grandparent-grandchild relationship, then it seems reasonable that that interest could ground claims to not have that relationship severed without good reason. This is in line with Draper’s argument. Before that relationship has been established, there may be a claim to allow it to form, and to support it, as that could be in the interests of both grandparents and grandchildren. This is in line with Overall’s argument. The former case, where the close relationship has already been established, is stronger than the latter, where the relationship has not yet formed (see also Shields forthcoming).

An interest in grandparenthood may add to the reasons why parents may choose fertility preservation for children, but that reason may not be legitimate in that specific context. For one, it treats the child as a means to an end (one’s grandparenthood aspirations). Second, even though some people may care about genetic relationships, this does not by itself ground any substantial claims on others – especially not others unable to consent to the course of action in question, in this case children facing cancer treatment. Should there be an interest in becoming a genetic grandparent specifically, considering all the substantial obstacles to making one’s own grandchildren – or having them made - there may be no legitimate way to act to make it happen. That interest, then, may not hold sufficient normative force to ground a claim.

## Closing thoughts

The path from a wish or an interest in becoming a grandparent to its fulfilment is full of thorns. This need not mean that there are no such wishes or interests, nor that they ought not to have a part to play. But it may mean that, all things considered, they may not generate strong enough claims, at least in cases such as those mentioned in the introduction of this paper.

If an interest in becoming a grandparent plays a role in reproductive decisions made ostensibly solely in the interests of those being reproduced, then this role needs to be made explicit, examined, and weighted against other relevant considerations. And if it turns out that there is such a thing as an interest in becoming a grandparent of a kind that generates claims against others, then we need to know what those claims are and how they play out in each case. There may be an interest in becoming a grandparent at the same time as there are significant obstacles in its way. You may lack the legitimate means to become, to make yourself a grandparent: your prima facie interest in becoming a grandparent may not be able to ground your becoming a grandparent. Once a grandchild exists, there may be fewer obstacles, and the claim to be allowed or supported in developing a relationship with one’s grandchild may be stronger. And a bond already formed may generate an even stronger claim.

## Footnotes

1 Where this is the case, parents may also have an interest in *not* becoming grandparents, or in limiting the number of grandchildren in their care. I thank Kristien Hens for suggesting this implication.

2 I thank Ingvar Johansson for suggesting this example.

## References

[CR1] Borelli, J. B., B. G. Bender, M. H. Puck, J. A. Salbenblatt, and A. Robinson. 1984. The meaning of early knowledge of a child’s infertility in families with 47,XXY and 45,X children. *Child Psychiatry and Human Development* 14:215–222. 6510028 10.1007/BF00706035

[CR2] Bourke, E. et al. 2014. A qualitative exploration of mothers’ and fathers’ experiences of having a child with Klinefelter syndrome and the process of reaching this diagnosis. *European Journal of Human Genetics* 22:18: 18–24. 23695282 10.1038/ejhg.2013.102 PMC3865389

[CR3] Brighouse, H., and A. Swift. 2014. *Family values. The ethics of Parent-Child relationships*. Princeton: Princeton University Press.

[CR4] Brownlee, K. 2020. *Being sure of each other. An essay on social rights and freedoms*. Oxford: Oxford University Press.

[CR5] Callanan, R. 2024. December Frozen legacy: The battle for posthumous parenthood in Ukraine. Available at: Accessed November 2025. https://www.gzeromedia.com/news/analysis/frozen-legacy-the-battle-for-posthumous-parenthood-in-ukraine.

[CR6] Dawson, B. 2024. December. A soldier was killed on active duty, and how his parents are fighting to use his sperm so they can have a grandchild, say reports. Available at Accessed November 2025. www.businessinsider.com/israel-parents-want-to-create-grandkids-dead-soldier-sons-sperm-2022-1.

[CR7] Dolin, G., D. E. Roberts, L. M. Rodriguez, and T. K. Woodruff. 2010. Medical hope, legal pitfalls: Potential legal issues in the emerging field of oncofertility. *Oncofertility. Cancer Treatment and Research* 156: 111–134. 10.1007/978-1-4419-6518-9_9 PMC2949971 20811829

[CR8] Draper, H. 2013. Grandparents’ entitlements and obligations. *Bioethics* 27 (6): 309–316. 23718643 10.1111/bioe.12028 PMC3770958

[CR9] EWFC 17. 2015. Application for an adoption order where the applicant had entered into a surrogacy arrangement with his mother.

[CR10] Fayemi, A. K. 2017. Family responsibilities and genetic disorders in Yoruba culture: The example of sickle cell anaemia. In *Parental responsibility in the context of neuroscience and genetics*, ed. K. Hens, and D. Cutas D Horstkötter. Dordrecht: Springer.

[CR12] Gheaus, A., G. Calder, and J. De Wispelaere. 2018. *Routledge Handbook on the Philosophy of Childhood and Children*. London: Routledge.

[CR11] Gheaus, A. 2011. Arguments for nonparental care for children. *Social Theory and Practice* 37 (3): 483–509.

[CR13] Golombok, S. 2020. *We are family. What really matters for parents and children*. London: Scribe.

[CR14] Guntram, L. 2021. May i have your uterus? The contribution of considering complexities preceding live uterus transplants. *Medical Humanities* 0: 1–13. 10.1136/medhum-2020-011864 PMC8639951 33627444

[CR15] Hashiloni-Dolev, Y. 2018. The effects if Jewish-Israeli family ideology on policy regarding reproductive technologies. In *Bioethics and biopolitics in Israel*, ed. H. Boas, et al. Cambridge University Press.

[CR16] Henderson, T. 2005. Grandparent visitation rights: Successful acquisition of court-ordered visitation. *Journal of Family Issues* 26 (1): 107–137.

[CR28] Hens, K. and D. Cutas. 2015. Preserving children’s fertility: two tales about children’s right to an open future and the margins of parental obligations. *Medicine, Health Care and Philosophy* 18 (2): 253–260. 10.1007/s11019-014-9596-3 10.1007/s11019-014-9596-3 25189425

[CR27] Hertz, R. and M. Nelson. 2019. *Random families. Genetic strangers, sperm donor siblings, and the creation of new kin*. Oxford: Oxford University Press.

[CR17] Johnson, E. K., I. Rosokija, and A. Shurba et al. 2017. Future fertility for individuals with differences of sex development: Parent attitudes and perspectives about decision-making. *Journal of Pediatric Urology* 13(4):402–413. 28713007 10.1016/j.jpurol.2017.06.002

[CR18] Kligman, G. 1988. *The wedding of the dead: Ritual, poetics, and popular culture in Transylvania*. University of California Press.

[CR19] Kornhaber, A. 1996. *Contemporary grandparenting*. London: Sage.

[CR20] Nisker, J., F. Baylis, and C. McLeod. 2006. Choice in fertility preservation in girls and adolescent women with cancer. *Cancer* 107 (7): 1606–1689. 10.1002/cncr.22106 16921478

[CR26] Nordqvist, P. and C. Smart. 2014.* Relative strangers: Family life, genes and donor conception*. New York: Palgrave.

[CR21] Overall, C. 2022. Ain’t love grand? Looking at grandparental love. In *Philosophy of love in the past, present, and future*, ed. A. Grahle and N. McKeever. Routledge.

[CR22] Pantea, M.C. 2011. Grandmothers as main caregivers in the context of parental migration. *European Journal of Social Work* 15 (1): 63–80.

[CR25] Savitsky, B. 2025. Reasons for opposition to posthumous reproduction and prior consent: Attitudes of Jewish men during the ongoing armed conflict. *Israel Journal of Health Policy Research* 14: 14–45. 10.1186/s13584-025-00703-6 PMC12278482 40685345

[CR23] Shields, L. *Beyond the parents: Justice in childrearing,* forthcoming.

[CR24] Smajdor, A. 2015. Perimortem gamete retrieval: Should we worry about consent? *Journal of Medical Ethics* 41(6):437–442. 24994620 10.1136/medethics-2013-101727

